# Case-control study on *CYP4B1* gene polymorphism and susceptibility to gastric cancer in the chinese Han population

**DOI:** 10.1186/s12920-022-01367-w

**Published:** 2022-10-28

**Authors:** Shuyong Yu, Zhuang Chen, Jiajia Cheng, Xingang Shi, Jiaqi Liu, Ping Zhong, Jian Song

**Affiliations:** 1 Department of Gastrointestinal Surgery, Hainan Cancer Hospital, #9, Changbinxisi Street, Xiuying District, Haikou City, Hainan Province China; 2 Department of Gastroenterology, Hainan Cancer Hospital, #9, Changbinxisi Street, Xiuying District, Haikou City, Hainan Province China; 3 Department of Gastroenterology, Changhai Hospital, Haikou City, China

**Keywords:** *CYP4B1* gene, Gastric cancer, Single nucleotide polymorphism, Rs4646491, Association

## Abstract

**Background:**

In China, gastric cancer (GC) is one of the most common malignant tumors. This study aimed to explore the relationship of rs2297810, rs4646491 and rs2297809 polymorphisms of *CYP4B1* with susceptibility to GC in the Chinese Han population.

**Methods:**

A case-control study including 707 GC cases and 707 normal controls was conducted. Three single nucleotide polymorphisms (SNPs) were genotyped by Agena MassARRAY system. Logistic regression analysis was utilized to assess the effects of SNPs on GC risk. Furthermore, multifactor dimensionality reduction (MDR) approach was used to analyze the SNP-SNP interactions.

**Results:**

No significant relationships were found between rs2297810 and rs2297809 and GC risk under all genetic models. For rs4646491, people with TC genotype had a 1.40-fold higher risk of GC than those with CC genotype (OR = 1.40; 95% CI = 1.13–1.74; *p* = 0.002), and people with TT-TC genotype had a 1.30-fold higher risk of GC than those with CC genotype (OR = 1.30; 95% CI = 1.06–1.61; *p* = 0.014). Stratification results showed that GC risk in people carrying TC genotype was higher than that in people with CC genotype, males (OR = 1.36; 95% CI = 1.06–1.75; *p* = 0.015), non-smokers (OR = 1.52; 95% CI = 1.11–2.07; *p* = 0.009) and non-drinkers (OR = 1.50; 95% CI = 1.10–2.04; *p* = 0.010). Additionally, the study also revealed that GC risk in people carrying TT-TC genotype was higher than that in people with CC genotype, males (OR = 1.29; 95% CI = 1.01–1.64; *p* = 0.040), non-smokers (OR = 1.40; 95% CI = 1.04–1.89; *p* = 0.027) and non-drinkers (OR = 1.39; 95% CI = 1.03–1.87; *p* = 0.030).

**Conclusion:**

This study firstly found that *CYP4B1*-rs4646491 was significantly correlated with GC risk, and it might be a risk factor for GC.

**Supplementary Information:**

The online version contains supplementary material available at 10.1186/s12920-022-01367-w.

## Introduction

Gastric cancer (GC) is the fifth most common cancer and the fourth leading cause of cancer death globally, according to the International Agency for Research on Cancer (IARC) in 2020 [1, 2]. Statistically, due to insignificant early symptoms, over 50% of GC patients are in advanced stage when diagnosed [3–5]. Modern research shows that the progression of GC is complex and diverse, and multiple factors contribute to its onset. Genetic factors, such as single nucleotide polymorphisms (SNPs), are considered to be inseparable from GC development[6]. The study of cancer-related gene polymorphisms may attribute to detect new predictive markers in cancer progression [7].

Cytochrome P450 family 4 (*CYP4*) enzymes are associated with biological functions such as skin barrier, inflammation, cardiovascular health, and cancer [8]. Variants of the *CYP4* gene have been shown to affect individual metabolic variations and disease susceptibility in previous studies [8]. *CYP4B1* is mainly expressed in the lungs [9] and has also been detected in adipose tissue, bladder, esophagus, stomach and so on (https://gtexportal.org/home/gene/CYP4B1). Studies have shown that mutation in the *CYP4B1* gene is considered to be a potentially cancer risk and may be involved in cancer by activating carcinogens, developing neovascularization and inducing inflammation [8, 10]. At present, more and more mutations in *CYP4B1* have been discovered by high-throughput sequencing technology. However, most of *CYP4B1* mutations remain undetected. Further studies of *CYP4B1* genetic variants are needed. The contribution of *CYP4B1* gene polymorphisms has been studied in other cancers, such as bladder cancer [11], urothelial cancer [12], and lung cancer [13]. However, relevant researches on the relationship between polymorphisms of the *CYP4B1* gene and GC are lacking.

Therefore, a case-control study was conducted to explore the relationship between the *CYP4B1* gene rs2297810, rs4646491 and rs2297809 polymorphisms and GC susceptibility. At the same time, by combining the clinical indicators and demographic characteristics (age, gender, smoking and drinking status), we could comprehensively investigated the association between potential influencing factors and the risk of GC. This study will provide a theoretical basis for the timely screening and diagnosis of GC.

## Materials and methods

### Study participants

The study recruited 707 healthy individuals and 707 GC patients from Hainan Cancer Hospital. All participants were genetically unrelated Han Chinese. Questionnaires and medical records were used to obtain clinical characteristics of participants. The average age of patients in this study (539 males and 168 females) was 59.48 ± 10.20 years. All patients with GC were diagnosed clinically and pathologically at their first stomach examination. The exclusion criteria for recruiting cases were: recurrent tumors or other malignant tumors; and patients underwent radiotherapy or chemotherapy. The average age of healthy individuals in this study (538 males and 169 females) was 59.34 ± 9.01 years. Healthy individuals had no history of cancer hereditary diseases. The Ethics Committee of Hainan Cancer Hospital provided its approval for this study. Each individual signed written informed consent.

### SNP selection and genotyping

The selection of SNP loci of the *CYP4B1* gene in our study was based on the following processes: First, all mutational loci of the *CYP4B1* gene were downloaded from the 1000 Genomes Project database. Second, Haploview software was applied to filter SNP loci based on specific parameters (Hardy-Weinberg equilibrium (HWE) > 0.01 and minor allele frequency (MAF) > 0.05). Finally, combined with primer design, three SNP loci (rs2297810, rs4646491 and rs2297809) were selected randomly. Peripheral blood (5 mL) from each subject was collected for DNA extraction by GoldMag genomic DNA purification kit (GoldMag Co. Ltd., Xi’an, China). The DNA concentration was estimated by NanoDrop 2000 (Thermo Scientific, Waltham, Massachusetts, USA).Ultimately, Agena MassARRAY system (Agena Bioscience, San Diego, CA, USA) was carried out to genotype SNPs.

### Statistical analysis

SPSS 20.0 was implemented for statistical analysis. All statistical tests were two-sided, and *p* < 0.05 was considered statistically significant. The *χ*^2^ test was used to evaluate the differences in demographic characteristics and genotype distribution of *CYP4B1* between cases and controls. Goodness-of-fit *χ*^2^ test was performed to test the HWE of SNPs in the control group. PLINK 1.9 (Harvard, Boston, MA, USA) was used to test the relationship between genotypes and GC risk in different genetic models. Odds ratios (ORs) and 95% confidence intervals (CIs) were obtained by logistic regression analysis after adjusting for age, gender, smoking and drinking status. Multifactor dimensionality reduction software (MDR version 3.0.2) was applied to explore the effects of SNP-SNP interactions. Additionally, one-way ANOVA was used to compare the genotype distribution of all clinical indicators of the case groups.

## Results

### Characteristics of study participants

Table 1 shows the demographic characteristics of GC patients and controls in this study, showing that there was no significant difference in the distribution of age (age > 60 or age ≤ 60) and gender (male or female) between the case group and control group (*p* = 0.264; *p* = 0.950). Smoking and drinking status revealed no significant difference between GC patients and control individuals (*p* = 0.958; *p* = 0.595). In the control group, the genotype frequency distribution of three SNPs satisfied HWE (*p* = 0.373; *p* = 0.488; *p* = 0.425; Table 2), implying that samples in this study were representative.

**Table 1:** Demographic characteristics of the cases and controls in gastric cancer

Characteristic		Case		Control		*p*
		**n**	**%**	**n**	**%**	
Total		707		707		
Age (Mean ± SD) years		59.48 ± 10.20		59.34 ± 9.01		0.793
	> 60	336	47.5	357	50.5	0.264
	≤ 60	371	52.5	350	49.5	
Gender	Male	539	76.2	538	76.1	0.950
	Female	168	23.8	169	23.9	
Smoking status	Yes	353	49.9	352	49.8	0.958
	No	354	50.1	355	50.2	
Alcohol consumption	Yes	353	49.9	343	48.5	0.595
	No	354	50.1	364	51.5	
Staging	III-IV	484	68.5			
	I-II	199	28.2			
	missing data	20	3.3			
lymphatic metastasis	Yes	477	67.5			
	No	178	25.2			
	missing data	52	7.3			

SD: Standard deviation; n: number.

*p* value < 0.05 indicates statistical significant.

**Table 2:** Primary information for *CYP4B1* gene polymorphisms in gastric cancer

SNP-ID	Chr, position	Gene	Alleles A/B	MAF		HWE-*p*	Role
				**Case**	**Control**		
rs2297810	1, 46,815,187	*CYP4B1*	A/G	0.258	0.254	0.373	SiPhy cons, Enhancer histone marks, Motifs changed, GRASP QTL hits, Selected Eqtl hits, dbSNP func annot
rs4646491	1, 46,815,212	*CYP4B1*	T/C	0.276	0.254	0.488	SiPhy cons, Enhancer histone marks, Motifs changed, GRASP QTL hits, Selected Eqtl hits, dbSNP func annot
rs2297809	1, 46,817,100	*CYP4B1*	T/C	0.254	0.252	0.425	SiPhy cons, Motifs changed, Selected Eqtl hits, dbSNP func annot

SNP: Single nucleotide polymorphism; Chr: chromosome; A: Minor alleles; B: Major alleles; MAF: Minor allele frequency; HWE: Hardy-Weinberg equilibrium.

*p* values were calculated from *χ*^2^ test (two sided).

### Association between three polymorphisms and the risk of GC

As shown in Table 3, the results proved that the genotype frequencies of *CYP4B1* gene rs2297810 and rs2297809 did not statistically different between in case group and control group (*p* > 0.05). Meanwhile, according to the logistic regression analysis, there was no relationship of *CYP4B1* gene rs2297810 and rs2297809 with the risk of GC. For *CYP4B1* gene rs4646491, the frequency distribution of the TC genotype was obviously different in two groups (*p* = 0.002), and the GC risk in the population with TC genotype was 1.40 times higher than those carrying CC genotype. The frequency distribution of the TT-TC genotype showed significant difference in two groups (*p* = 0.014), and the GC risk in the population carrying TT-TC genotype was 1.30 times higher than those with CC genotype. Taken together, TC genotype and TT-TC genotype could be dangerous elements for GC. However, we did not observe a significant association of the rs4646491 polymorphism with GC risk in allelic, recessive and additive models (*p* > 0.05; Table 3).

**Table 3:** Genetic model analyses of the association between *CYP4B1* polymorphisms and the risk of gastric cancer (adjusted for gender, age, smoking and drinking)

SNP-ID	Model	Genotype	Case		Control		Adjusted by gender, age, smoking and drinking	
			**n**	**%**	**n**	**%**	**OR (95% CI)**	***p***
rs2297810	allele	A	364	25.82	359	25.39	1.02 (0.86–1.21)	0.795
		G	1046	74.18	1055	74.61	1.00	
	genotype	AA	43	6.10	50	7.07	0.89 (0.58–1.37)	0.595
		AG	278	39.43	259	36.63	1.11 (0.89–1.39)	0.340
		GG	384	54.47	398	56.30	1.00	
	dominant	AA-AG	321	45.53	309	43.71	1.08 (0.87–1.33)	0.490
		GG	384	54.47	398	56.29	1.00	
	recessive	AA	43	6.10	50	7.07	0.85 (0.56–1.30)	0.456
		AG-GG	662	93.90	657	92.93	1.00	
	additive	---	---	---	---	---	1.02 (0.86–1.21)	0.798
rs4646491	allele	T	388	27.60	359	25.39	1.12 (0.95–1.32)	0.184
		C	1018	72.40	1055	74.61	1.00	
	genotype	TT	34	4.84	49	6.93	0.79 (0.50–1.25)	0.309
		TC	320	45.52	261	36.92	1.40 (1.13–1.74)	**0.002**
		CC	349	49.64	397	56.15	1.00	
	dominant	TT-TC	354	50.36	310	43.85	1.30 (1.06–1.61)	**0.014**
		CC	349	49.64	397	56.15	1.00	
	recessive	TT	34	4.84	49	6.93	0.68 (0.43–1.07)	0.094
		TC-CC	669	95.16	658	93.07	1.00	
	additive	---	---	---	---	---	1.13 (0.95–1.34)	0.167
rs2297809	allele	T	359	25.42	356	25.25	1.01 (0.09–0.85)	0.914
		C	1053	74.58	1054	74.75	1.00	
	genotype	TT	38	5.38	49	6.95	0.80 (0.51–1.25)	0.324
		TC	283	40.08	258	36.60	1.14 (0.91–1.42)	0.253
		CC	385	54.54	398	56.45	1.00	
	dominant	TT-TC	321	45.47	307	43.55	1.08 (0.88–1.34)	0.460
		CC	385	54.53	398	56.45	1.00	
	recessive	TT	38	5.38	49	6.95	0.76 (0.49–1.18)	0.215
		TC-CC	668	94.62	656	93.05	1.00	
	additive	---	---	---	---	---	1.01 (0.85–1.20)	0.911

SNP: single nucleotide polymorphism; n: number; OR: Odds ratio; 95% CI: 95% Confidence interval.

*p* < 0.05 indicates statistical significance.

### Stratified analysis

Table 4 presents the stratification results of the relationship between rs2297810, rs4646491 and rs2297809 polymorphisms of *CYP4B1* gene and the risk of GC. More precisely, stratification by age revealed that the frequency distributions of TC heterozygous genotype and TT-TC genotype of rs4646491 were statistically different between the case and control groups (*p* = 0.015; *p* = 0.040). GC risk in TC genotype carriers was 1.36 times higher than that in CC genotype carriers, and the risk in TT-TC genotype carriers was 1.29 times than that in CC genotype carriers. Stratification of participants according to their smoking habits indicated that the frequency distribution of TC heterozygous genotype and TT-TC genotype of rs4646491 were statistically different between the cases and controls (*p* = 0.009; *p* = 0.027). The risk of GC in people with TC genotype was 1.52 times higher than those with CC genotype, and the risk in people with TT-TC genotype was 1.40 times than those with CC genotype. Furthermore, in the non-drinking subgroup, the frequency distribution of TC heterozygous genotype and TT-TC genotype of rs4646491 were statistically different between cases and controls (*p* = 0.010; *p* = 0.030). The risk of GC in people with TC genotype was 1.50 times higher than those with CC genotype, and the risk in people with TT-TC genotype was 1.39 times than those with CC genotype. The rs4646491 polymorphism TC and TT-TC genotype might be risk factors for GC. In the allele, recessive and additive models, the frequency distributions of rs4646491 genotypes were not statistically different (*p* > 0.05). For rs2297810 and rs2297809, no significant differences were observed in each group under all genetic models. Age-, stage-, and lymphatic metastasis-stratified analyses were also performed to investigate the association between SNPs and GC risk under different genotypic models, and no significant differences were found (Supplementary Table 1).

**Table 4:** Stratified analyses between SNPs on *CYP4B1* and gastric cancer risk by gender, smoking and alcohol under different genotypic models

SNP-ID	Model	Genotype	Gender stratification				Smoking stratification				Drinking stratification			
			**Males**		**Females**		**Yes**		**No**		**Yes**		**No**	
			**OR (95% CI)**	***p***	**OR (95% CI)**	***p***	**OR (95% CI)**	***p***	**OR (95% CI)**	***p***	**OR (95% CI)**	***p***	**OR (95% CI)**	***p***
rs2297810	allele	A	1.03 (0.85–1.25)	0.748	0.99 (0.70–1.41)	0.965	1.03 (0.81–1.31)	0.830	1.02 (0.80–1.29)	0.879	1.04 (0.81–1.32)	0.783	1.01 (0.80–1.28)	0.918
		G	1.00		1.00		1.00		1.00		1.00		1.00	
	genotype	AA	0.91 (0.55–1.51)	0.714	0.95 (0.40–2.25)	0.909	0.89 (0.48–1.66)	0.722	0.90 (0.49–1.64)	0.726	0.94 (0.50–1.78)	0.860	0.90 (0.49–1.63)	0.717
		AG	1.11 (0.87–1.43)	0.405	1.17 (0.73–1.86)	0.514	1.12 (0.82–1.53)	0.481	1.15 (0.84–1.57)	0.387	1.14 (0.83–1.57)	0.403	1.13 (0.83–1.54)	0.443
		GG	1.00		1.00		1.00		1.00		1.00		1.00	
	dominant	AA-AG	1.08 (0.85–1.38)	0.522	1.13 (0.73–1.75)	0.592	1.08 (0.80–1.46)	0.600	1.11 (0.82–1.49)	0.509	1.11 (0.82–1.51)	0.486	1.09 (0.81–1.47)	0.569
		GG	1.00		1.00		1.00		1.00		1.00		1.00	
	recessive	AA	0.87 (0.53–1.43)	0.583	0.90 (0.39–2.07)	0.798	0.85 (0.46–1.57)	0.610	0.85 (0.47–1.53)	0.587	0.89 (0.48–1.66)	0.723	0.85 (0.47–1.53)	0.593
		AG-GG	1.00		1.00		1.00		1.00		1.00		1.00	
	additive	---	1.03 (0.85–1.25)	0.763	1.06 (0.75–1.49)	0.753	1.03 (0.81–1.31)	0.825	1.04 (0.82–1.32)	0.757	1.05 (0.83–1.35)	0.672	1.03 (0.81–1.30)	0.811
rs4646491	allele	T	1.13 (0.93–1.36)	0.222	1.10 (0.78–1.56)	0.594	1.09 (0.86–1.38)	0.486	1.15 (0.91–1.46)	0.238	1.09 (0.86–1.38)	0.488	1.15 (0.91–1.46)	0.233
		C	1.00		1.00		1.00		1.00		1.00		1.00	
	genotype	TT	0.86 (0.50–1.46)	0.567	0.69 (0.26–1.82)	0.454	0.77 (0.40–1.48)	0.432	0.82 (0.43–1.58)	0.551	0.81 (0.42–1.57)	0.531	0.82 (0.43–1.57)	0.547
		TC	1.36 (1.06–1.75)	**0.015**	1.57 (0.99–2.48)	0.055	1.33 (0.98–1.82)	0.069	1.52 (1.11–2.07)	**0.009**	1.36 (0.99–1.86)	0.054	1.50 (1.10–2.04)	**0.010**
		CC	1.00		1.00		1.00		1.00		1.00		1.00	
	dominant	TT-TC	1.29 (1.01–1.64)	**0.040**	1.40 (0.90–2.18)	0.132	1.24 (0.92–1.68)	0.151	1.40 (1.04–1.89)	**0.027**	1.28 (0.94–1.73)	0.115	1.39 (1.03–1.87)	**0.030**
		CC	1.00		1.00		1.00		1.00		1.00		1.00	
	recessive	TT	0.75 (0.45–1.26)	0.272	0.58 (0.22–1.49)	0.255	0.68 (0.36–1.29)	0.237	0.69 (0.36–1.30)	0.247	0.71 (0.37–1.36)	0.301	0.69 (0.36–1.29)	0.244
		TC-CC	1.00		1.00		1.00		1.00		1.00		1.00	
	additive	---	1.14 (0.93–1.39)	0.202	1.15 (0.81–1.65)	0.436	1.10 (0.86–1.40)	0.468	1.19 (0.93–1.52)	0.172	1.12 (0.87–1.44)	0.367	1.18 (0.92–1.50)	0.185
rs2297809	allele	T	1.01 (0.83–1.22)	0.942	1.02 (0.71–1.45)	0.928	0.97 (0.76–1.23)	0.806	1.05 (0.83–1.33)	0.691	1.01 (0.79–1.29)	0.926	1.01 (0.80–1.28)	0.945
		C	1.00		1.00		1.00		1.00		1.00		1.00	
	genotype	TT	0.86 (0.51–1.44)	0.559	0.73 (0.29–1.85)	0.505	0.77 (0.41–1.46)	0.430	0.83 (0.44–1.56)	0.563	0.88 (0.46–1.66)	0.685	0.77 (0.41–1.45)	0.420
		TC	1.09 (0.85–1.40)	0.508	1.41 (0.88–2.25)	0.151	1.07 (0.78–1.46)	0.692	1.26 (0.92–1.72)	0.152	1.14 (0.83–1.57)	0.417	1.19 (0.87–1.61)	0.282
		CC	1.00		1.00		1.00		1.00		1.00		1.00	
	dominant	TT-TC	1.05 (0.83–1.34)	0.668	1.28 (0.82–1.99)	0.283	1.02 (0.76–1.37)	0.899	1.19 (0.88–1.60)	0.265	1.10 (0.81–1.49)	0.544	1.12 (0.83–1.50)	0.461
		CC	1.00		1.00		1.00		1.00		1.00		1.00	
	recessive	TT	0.83 (0.50–1.37)	0.465	0.64 (0.26–1.59)	0.335	0.75 (0.40–1.41)	0.376	0.76 (0.41–1.41)	0.378	0.83 (0.45–1.55)	0.563	0.72 (0.38–1.34)	0.297
		TC-CC	1.00		1.00		1.00		1.00		1.00		1.00	
	additive	---	1.01 (0.83–1.23)	0.948	1.09 (0.76–1.55)	0.636	0.97 (0.76–1.24)	0.806	1.07 (0.84–1.37)	0.579	1.03 (0.81–1.32)	0.791	1.02 (0.80–1.30)	0.850

SNP: single nucleotide polymorphism; OR: Odds ratio; 95% CI: 95% Confidence interval.

*p* < 0.05 indicates statistical significance.

### MDR analysis on the correlation between SNP-SNP interactions and the risk of GC

**Fig. 1:**
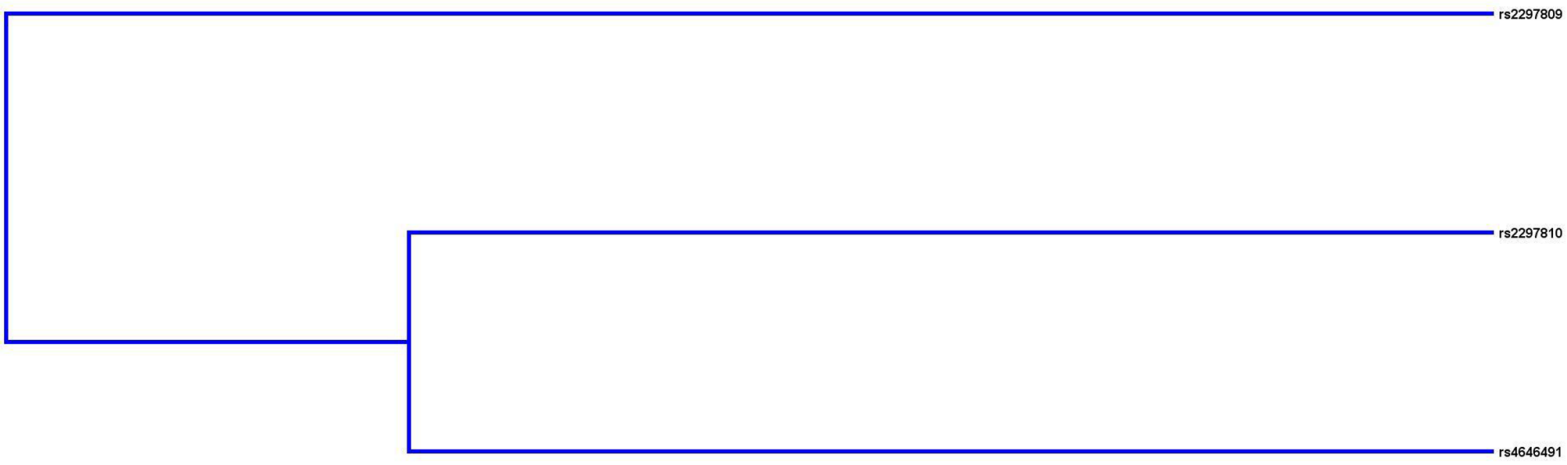
Interaction dendrogram by MDR analysis of three SNPs on *CYP4B1*. Blue connections indicate redundancy or lack of synergistic interactions among markers

The impact of SNP-SNP high-order interactions on the risk of GC was assessed by MDR analysis. The dendrogram (Fig. 1) indicates that the interaction between rs2297810 and rs4646491 had a strong antagonistic effect on the *CYP4B1* gene. The best locus model of *CYP4B1* SNPs for predicting GC susceptibility is shown in Table 5. Among all models, the two-locus model (rs2297810-rs4646491) was the best predictive model with a perfect cross-validation consistency (CVC) of 10/10 and a test accuracy of 0.547 (OR = 1.49, 95% CI = 1.21–1.84, *p* < 0.001).

**Table 5:** Summary of SNP-SNP interactions on the risk of gastric cancer analyzed by MDR method

Model	Bal.Acc. Training	Bal.Acc. Testing	CVC	OR (95% CI)	*p*
rs4646491	0.545	0.545	10/10	1.44 (1.17–1.79)	**< 0.001**
rs2297810,rs4646491*	0.549	0.547	10/10	1.49 (1.21–1.84)	**< 0.001**
rs2297810,rs4646491,rs2297809	0.551	0.540	10/10	1.52 (1.23–1.88)	**< 0.001**

MDR: multifactor dimensionality reduction; Bal.Acc: balanced accuracy; CVC: cross-validation consistency; OR: odds ratio; 95%CI: 95% confidence interval.

*p* < 0.05 indicates statistical significance.

*: The best model in MDR analysis.

### Different clinical indicators based on the genotypes of selected SNPs

We also analyzed the association between three SNPs and clinical indicators of GC patients. Clinical indicators included carcinoma embryonic antigen (CEA), alpha fetoprotein (AFP), carbohydrate antigen 199 (CA199), carbohydrate antigen 125 (CA125) and carbohydrate antigen 50 (CA50). The results showed no significant association between clinical indicators of three SNPs and the risk of GC under different genotypes (Supplementary Table 2).

## Discussion

At present, the incidence of GC shows a gradual upward trend. It has been reported that various factors may lead to the occurrence of GC, including inflammation, infection, diet, environmental factors, immune factors and genetic factors. Cytochrome P450 (CYP) is a superfamily of enzymes and a typical phase I drug metabolism enzyme, which is located in the inner membrane of mitochondria and the endoplasmic reticulum membrane of eukaryotic cells. [8, 14]. *CYP4B1* is a member of the CYP4B subfamily, and the current researches on *CYP4B1* gene mainly focus on its function analysis. Studies have shown that *CYP4B1* plays an important role in the metabolic process of endogenous compounds and exogenous substances (drugs, environmental toxicants, pro-carcinogens), thereby affecting the occurrence and development of diseases [15]. It is well known that the drug metabolizing enzymes in the human body are also the metabolic enzymes of important carcinogens, which control and affect the metabolism of carcinogens, so they play a decisive role in tumor susceptibility. The stomach is the major site of exposure to poisons and carcinogens, and its metabolic balance is crucial for maintaining normal physiological functions. Therefore, we have reason to speculate that *CYP4B1* is closely related to the pathogenic mechanism of GC. Based on this, our study investigated the association of SNPs (rs2297810, rs4646491 and rs2297809) in the *CYP4B1* gene with GC risk. As a result, we found for the first time that the TC genotype of *CYP4B1*-rs4646491 was statistically associated with an increased risk of GC. Our findings can provide a new perspective for future research on GC.

In recent years, the study of SNP has become an effective tool and method for molecular genetics research, and has attracted much attention [16]. In our study, three SNPs (rs2297810, rs4646491 and rs2297809) of the *CYP4B1* gene were taken as the research objects to explore the relationship between them and GC risk. As a result, we found that only rs4646491 was associated with increased GC risk. Notably, no studies describing the role of rs4646491 in disease susceptibility have been published previously. As for rs2297809 and rs2297810 polymorphisms, only a few studies have reported them. For example, rs2297809 and rs2297810 polymorphisms were reported in a study exploring genes controlling the age at onset of Parkinson’s disease, but no positive results were observed [17]. Likewise, our findings also showed that rs2297809 and rs2297810 polymorphisms were not significantly associated with GC risk under all genetic models. Current evidence may suggest that rs2297810 and rs2297809 are not associated with GC risk, but lager sample size and further validation in other populations are need.

It has been reported that there is a gender difference in the incidence of GC worldwide, and the incidence of GC in men is about twice as high as that in women, especially in countries with a high incidence of GC [18]. We got a similar result by stratification analysis that *CYP4B1* gene rs4646491 was associated with the risk of GC in the male group. On the one hand, we believed that men’s unhealthy lifestyle habits (smoking or drinking) contribute to this fact. On the other hand, this may be related to the role of androgens in regulating *CYP4B1* expression. Imaoka S et al. [20] have found that the expression of *CYP4B1* in the bladder of mature male rats is higher than that in the bladder of mature female rats, and its expression increases with the growth of rat, suggesting that the *CYP4B1* gene may be specifically expressed in males. Combined with our findings that the rs4646491 polymorphism was associated with an increased risk of GC in men, further indicating that androgens may affect the expression of *CYP4B1* by affecting the mutation of rs4646491, thereby affecting the occurrence and development of GC.

Smoking and alcohol consumption are major risk factors for GC. Previous studies have shown that risk of GC is higher in smokers and drinkers when compared to no-smokers and no-drinkers [19–23]. However, studies have also found that non-drinkers [24] and non-smokers are still at the risk of developing GC [4]. Our results manifested that there was a correlation between the lifestyle of non-smokers and non-drinkers and the risk of GC. This was partially consistent with our findings. As is known to all, tumor-related clinical indicators is of great significance for the early detection, diagnosis and prognosis monitoring of tumors. Many studies have shown that CEA [25–27], AFP [28], CA199 [25], CA125 [25] and CA50 [29] are common biomarkers in GC. However, no significant association between clinical indicators (CEA, AFP, CA19-9, CA125 and CA50) of targeted SNPs and the risk of GC under different genotypes was found in our study. The reason for this result may be related to the small random sample in our study. In follow-up studies, the sample size should be expanded for more in-depth research.

This research firstly showed a significant association between rs4646491 on *CYP4B1* gene and GC risk, suggesting that rs4646491 polymorphism on *CYP4B1* was an important factor for predicting the risk of GC, and it was beneficial for the early discovery and diagnosis of GC. At the same time, there are several limitations in the study. Firstly, GC patients and healthy subjects from the same hospital may not be representative of the population as a whole. Secondly, this study includes only three SNPs, which may not provide a comprehensive understanding of genetic variations in the *CYP4B1* gene. Therefore, further studies are needed to explore the association of multiple SNPs in the *CYP4B1* gene with GC risk in populations from different regions and ethnicities.

## Conclusion

In conclusion, our study firstly indicates that the rs4646491 polymorphism on *CYP4B1* may be associated with the risk of GC. This study will provide a theoretical basis for the early detection and treatment of GC in the future.

## Electronic supplementary material

Below is the link to the electronic supplementary material.

Supplementary Material 1

## Data Availability

The datasets supporting the conclusions of this article are included within the article and its additional files.

## Abbreviations

GC: Gastric cancer.
SNPs: Single nucleotide polymorphisms.
CYP: Cytochrome P450.
MDR: Multifactor dimensionality reduction.
CYP4: Cytochrome P450 family 4.
HWE: Hardy-Weinberg equilibrium.
MAF: Minor allele frequency.
CEA: Carcinoma embryonic antigen.
AFP: Alpha fetoprotein.
CA199: Carbohydrate antigen 199.
CA125: Carbohydrate antigen 125.
CA50: Carbohydrate antigen 50.
