# Supplementary material for: Case-control study on CYP4B1 gene polymorphism and susceptibility to gastric cancer in the chinese Han population
Source: BMC Med Genomics. 2022 Oct 28;15:223. doi: 10.1186/s12920-022-01367-w (PMC9615408; doi:10.1186/s12920-022-01367-w)
Supplement: Supplementary file 1 — Supplementary Material 1 [file 12920_2022_1367_MOESM1_ESM.docx]

**Supplementary table 1 Stratified analyses between SNPs on *CYP4B1* gene and gastric cancer risk by age, stage and lymphatic metastasis under different genotypic models**

| **SNP-ID** | **Model** | **Genotype** | **Age stratification in case vs controls** | | | | **Stage III-IV vs I-II** | | **lymphatic metastasis Yes vs No** | |
| --- | --- | --- | --- | --- | --- | --- | --- | --- | --- | --- |
|  |  |  | **> 60** | | **≤ 60** | |  | |  | |
|  |  |  | **OR (95% CI)** | ***p*** | **OR (95% CI)** | ***p*** | **OR (95% CI)** | ***p*** | **OR (95% CI)** | ***p*** |
| rs2297810 | allele | A | 1.12 (0.88-1.42) | 0.359 | 0.94 (0.74-1.20) | 0.627 | 0.81 (0.63-1.06) | 0.121 | 0.93 (0.71-1.23) | 0.623 |
|  |  | G | 1.00 |  | 1.00 |  | 1.00 |  | 1.00 |  |
|  | genotype | AA | 0.87 (0.47-1.59) | 0.648 | 1.00 (0.53-1.90) | 0.993 | 0.73 (0.37-1.46) | 0.375 | 1.26 (0.57-2.75) | 0.567 |
|  |  | AG | 1.42 (1.03-1.96) | 0.032 | 0.89 (0.65-1.21) | 0.443 | 0.76 (0.54-1.08) | 0.128 | 0.78 (0.54-1.12) | 0.181 |
|  |  | GG | 1.00 |  | 1.00 |  | 1.00 |  | 1.00 |  |
|  | dominant | AA-AG | 1.31 (0.97-1.78) | 0.081 | 0.90 (0.67-1.21) | 0.490 | 0.76 (0.54-1.06) | 0.107 | 0.83 (0.58-1.18) | 0.296 |
|  |  | GG | 1.00 |  | 1.00 |  | 1.00 |  | 1.00 |  |
|  | recessive | AA | 0.75 (0.42-1.36) | 0.344 | 1.05 (0.56-1.98) | 0.870 | 0.83 (0.42-1.62) | 0.576 | 1.40 (0.65-3.01) | 0.387 |
|  |  | AG-GG | 1.00 |  | 1.00 |  | 1.00 |  | 1.00 |  |
|  | additive | --- | 1.13 (0.89-1.44) | 0.322 | 0.94 (0.74-1.20) | 0.616 | 0.81 (0.62-1.06) | 0.125 | 0.93 (0.70-1.23) | 0.617 |
| rs4646491 | allele | T | 1.19 (0.94-1.51) | 0.146 | 1.06 (0.84-1.34) | 0.629 | 0.82 (0.64-1.06) | 0.137 | 0.96 (0.73-1.25) | 0.745 |
|  |  | C | 1.00 |  | 1.00 |  | 1.00 |  | 1.00 |  |
|  | genotype | TT | 0.82 (0.44-1.54) | 0.543 | 0.87 (0.43-1.74) | 0.687 | 0.58 (0.28-1.23) | 0.153 | 1.31 (0.54-3.16) | 0.549 |
|  |  | TC | 1.70 (1.23-2.34) | 0.001 | 1.17 (0.87-1.59) | 0.303 | 0.81 (0.58-1.15) | 0.248 | 0.83 (0.58-1.18) | 0.300 |
|  |  | CC | 1.00 |  | 1.00 |  | 1.00 |  | 1.00 |  |
|  | dominant | TT-TC | 1.52 (1.12-2.07) | 0.007 | 1.14 (0.85-1.53) | 0.394 | 0.79 (0.56-1.10) | 0.165 | 0.86 (0.61-1.23) | 0.410 |
|  |  | CC | 1.00 |  | 1.00 |  | 1.00 |  | 1.00 |  |
|  | recessive | TT | 0.66 (0.36-1.21) | 0.178 | 0.81 （0.41-1.61） | 0.541 | 0.64 (0.31-1.33) | 0.232 | 1.44 (0.61-3.40) | 0.410 |
|  |  | TC-CC | 1.00 |  | 1.00 |  | 1.00 |  | 1.00 |  |
|  | additive | --- | 1.22 (0.96-1.56) | 0.108 | 1.07 (0.83-1.37) | 0.618 | 0.79 (0.60-1.05) | 0.104 | 0.94 (0.70-1.27) | 0.696 |
| rs2297809 | allele | T | 1.07 (0.84-1.36) | 0.585 | 0.96 (0.76-1.22) | 0.742 | 0.83 (0.64-1.08) | 0.174 | 0.89 (0.68-1.17) | 0.416 |
|  |  | C | 1.00 |  | 1.00 |  | 1.00 |  | 1.00 |  |
|  | genotype | TT | 0.70 (0.37-1.31) | 0.267 | 1.04 (0.53-2.03) | 0.920 | 0.77 (0.37-1.61) | 0.492 | 1.20 (0.52-2.74) | 0.669 |
|  |  | TC | 1.43 (1.04-1.98) | 0.029 | 0.91 (0.67-1.24) | 0.561 | 0.77 (0.54-1.09) | 0.135 | 0.73 (0.51-1.05) | 0.092 |
|  |  | CC | 1.00 |  | 1.00 |  | 1.00 |  | 1.00 |  |
|  | dominant | TT-TC | 1.28 (0.94-1.74) | 0.111 | 0.93 (0.69-1.25) | 0.614 | 0.77 (0.55-1.08) | 0.123 | 0.77 (0.55-1.10) | 0.153 |
|  |  | CC | 1.00 |  | 1.00 |  | 1.00 |  | 1.00 |  |
|  | recessive | TT | 0.61 (0.33-1.12) | 0.110 | 1.08 (0.56-2.08) | 0.831 | 0.87 (0.43-1.78) | 0.704 | 1.38 (0.61-3.09) | 0.441 |
|  |  | TC-CC | 1.00 |  | 1.00 |  | 1.00 |  | 1.00 |  |
|  | additive | --- | 1.08 (0.85-1.38) | 0.543 | 0.96 (0.75-1.23) | 0.734 | 0.82 (0.62-1.08) | 0.155 | 0.88 (0.66-1.17) | 0.375 |
| SNP: single nucleotide polymorphism; OR: Odds ratio; 95% CI: 95% Confidence interval; HWE: Hardy-Weinberg equilibrium.  *p* < 0.05 indicates statistical significance. | | | | | | | | | | |

| **Supplementary table 2 Difference of clinical indicators based on the genotypes of selected SNPs on *CYP4B1* gene in gastric cancer** | | | | | | | |
| --- | --- | --- | --- | --- | --- | --- | --- |
| **Characteristics** | **rs2297810** | | | | | | |
|  | **n** | **AA** | **n** | **GG** | **n** | **GA** | ***p*** |
| CEA (ng/ml) | 20 | 9.73 ± 5.83 | 205 | 20.70 ± 77.49 | 163 | 13.20 ± 36.3 | 0.436 |
| AFP (ng/ml) | 20 | 8.50 ± 4.25 | 189 | 8.11 ± 8.78 | 158 | 8.65 ± 12.34 | 0.886 |
| CA199 (U/ml) | 17 | 23.84 ± 23.83 | 177 | 27.21 ± 41.05 | 140 | 31.86 ± 79.58 | 0.740 |
| CA125 (U/ml) | 4 | 11.25 ± 9.15 | 92 | 23.74 ± 32.50 | 85 | 23.73 ± 30.43 | 0.733 |
| CA50 (U/ml) | 17 | 5.37 ± 6.36 | 102 | 6.16 ± 9.02 | 78 | 8.71 ± 14.33 | 0.262 |
|  | **rs4646491** | | | | | | |
|  | **n** | **TT** | **n** | **CC** | **n** | **CT** | ***p*** |
| CEA (ng/ml) | 17 | 9.83 ± 6.25 | 184 | 21.63 ± 81.68 | 186 | 13.11 ± 34.16 | 0.361 |
| AFP (ng/ml) | 16 | 8.87 ± 4.42 | 170 | 8.36 ± 10.33 | 180 | 8.33 ± 10.68 | 0.980 |
| CA199 (U/ml) | 14 | 24.37 ± 25.95 | 156 | 24.21 ± 37.72 | 164 | 34.01 ± 76.41 | 0.327 |
| CA125 (U/ml) | 4 | 11.25 ± 9.15 | 85 | 23.36 ± 30.56 | 91 | 24.32 ± 32.47 | 0.714 |
| CA50 (U/ml) | 13 | 4.94 ± 5.99 | 89 | 6.71 ± 9.9 | 95 | 7.76 ± 12.98 | 0.637 |
|  | **rs2297809** | | | | | | |
|  | **n** | **TT** | **n** | **TC** | **n** | **CC** | ***p*** |
| CEA (ng/ml) | 19 | 9.92 ± 5.93 | 163 | 13.28 ± 36.29 | 206 | 20.56 ± 77.32 | 0.461 |
| AFP (ng/ml) | 19 | 8.34 ± 4.31 | 158 | 8.80 ± 12.34 | 190 | 8.00 ± 8.74 | 0.773 |
| CA199 (U/ml) | 16 | 24.83 ± 24.26 | 142 | 32.93 ± 79.79 | 177 | 26.40 ± 39.83 | 0.599 |
| CA125 (U/ml) | 4 | 11.25 ± 9.15 | 84 | 23.64 ± 30.66 | 93 | 23.82 ± 32.28 | 0.732 |
| CA50 (U/ml) | 16 | 5.41 ± 6.57 | 79 | 9.04 ± 14.45 | 102 | 5.86 ± 8.66 | 0.141 |
| CEA: carcinoma embryonic antigen; AFP: alpha fetoprotein; CA199: carbohydrate antigen 199; CA125: carbohydrate antigen 125; CA50: carbohydrate antigen 50.  *p*<0.05 indicates statistical significance. | | | | | | | |
